# Development of a Digital Health Intervention for Rheumatoid Arthritis Symptom Management in a Biotechnology Industry Context: Protocol for the Application of a Human-Centered Design Framework

**DOI:** 10.2196/16430

**Published:** 2022-03-22

**Authors:** Lisa Nugent, Robin Anthony Kouyate, Shawna Jackson, Meredith Y Smith

**Affiliations:** 1 Amgen Thousand Oaks, CA United States

**Keywords:** human-centered design, patient-reported outcomes, rheumatoid arthritis, digital journal, patient diaries, data visualization, mobile phone

## Abstract

**Background:**

Involving chronically ill patients in the management of their health is widely recognized as a vital component of high-quality health care. However, to assume the role of informed participants, patients need both access to their health information and assistance in interpreting such data. Smartphone technology with SMS text messaging functionality offers a convenient and minimally demanding mechanism for providing such dual capabilities to patients. To date, a number of similar digital tools have been developed for use in various chronic and progressive disease conditions, including rheumatoid arthritis.

**Objective:**

This paper aims to describe the development of a research protocol that applies a human-centered design (HCD) approach to develop a mobile health (mHealth) intervention to support symptom management and treatment adherence for rheumatoid arthritis.

**Methods:**

To guide the development of the mHealth intervention for use within a commercial biotechnology context, we selected and applied an HCD framework consisting of three phases: understanding, ideation, and implementation.

**Results:**

Leveraging the framework, we mapped the key objectives and research questions to each phase and identified the HCD techniques and methods most suitable for addressing them. In addition, we identified the need to include a fourth phase, one that referred to postimplementation assessment, which would enable evaluation of patient engagement and intervention impact on symptom self-management.

**Conclusions:**

This paper presents a research protocol that applied an HCD framework to guide the development of an mHealth intervention within a commercial biotechnology context. This type of guidance is salient because commercial entities are becoming one of the leading producers of this type of intervention. However, the methodologies used and challenges faced from a research and development perspective are not well-represented in the published research literature to date. Our application of the HCD framework yielded important findings. Each phase of the HCD framework provided important guidance for increasing the likelihood that the final product would be understandable, acceptable, feasible, and engaging to use. Consistent with other researchers in the field of mHealth interventions, we identified the need to add a fourth phase to the HCD framework, one that focused on a postimplementation assessment to guide further improvements to support adoption in real-world settings.

**International Registered Report Identifier (IRRID):**

RR1-10.2196/16430

## Introduction

### Background

Patient-centeredness is increasingly recognized as a hallmark of high-quality health care delivery and drug development [1]. Empowering patients to manage their own health and disease conditions is a key aspect of patient-centeredness, which has been shown to improve treatment adherence and other patient outcomes [2]. However, to assume the role of empowered participants, patients need access to their health data and assistance in interpreting it. Smartphone technology with SMS text messaging functionality offers a convenient and minimally demanding mechanism for providing such dual capabilities to patients. To date, numerous types of digital tools have been developed for use in various chronic and progressive disease conditions, including for rheumatoid arthritis (RA).

RA is a chronic, progressive autoimmune disease. In the United States, an estimated 41 in 100,000 adults are diagnosed with this condition annually [3,4]. Typically, patients with this disease are older, White, and female [5]. Hallmarks of RA include functional disabilities that increase in severity over time and premature mortality [6,7]. Key disease markers include morning stiffness, pain, and fatigue. Treatments for RA feature a range of disease-modifying therapies, including biologics [6]. However, long-term adherence to therapy, especially biologic regimens has been shown to be poor [6,8]. Multiple factors contribute to nonadherence, including limited patient awareness and understanding of the disease, how it progresses, and how different treatments affect symptom expression over time [9,10].

Over the last two decades, patient-reported outcome measures (PROMs) have been developed to capture important aspects of RA disease and treatment [11]. Increasingly, static, one-time measurements have been replaced by a more dynamic approach in which PROMs are assessed at defined time points over a 24-hour period [11]. This type of periodic assessment is especially useful for evaluating disease progression and treatment impact and provides data that are informative for both health care professionals (HCPs) and patients alike [12]. To date, PROMs have been used predominantly in the clinical trial context. However, there is a growing recognition of their potential value in routine rheumatologic outpatient care as well [7]. Patient diaries have long been used to collect patient-reported outcomes (PROs), including RA-related symptoms [13]. Increasingly, digital diaries have supplanted paper-based versions largely because of the superior ease and timeliness of data capture [12,14]. Sharing the results of these assessments with patients visually in a graphical format has been shown to improve treatment adherence, increase patient trust in their physician, enhance patient-provider communication, boost patients’ disease coping capability, and improve their understanding of the effects of disease activity and treatment [13].

Although the use of such electronic (*eHealth*) interventions has been growing, sustaining their use has remained elusive. A major contributing factor in this regard is that interventions have typically been developed with little to no involvement from the patient [13]. This lack of patient-centeredness has led to persistent usability problems and high attrition rates, resulting in mobile health (mHealth) interventions that are *high tech with a low impact* [15,16].

Currently, the peer-reviewed research literature describing patient-centered interventions to support RA symptom management and treatment adherence, digital or otherwise, is sparse [14]. Azevedo et al [17] used a patient-centered approach to develop a smartphone app for RA self-management. The study consisted of a cross-sectional patient survey to assess the usefulness of the app in supporting RA self-management, preferred features, and the degree to which patients would be willing to use and pay for it. However, no details were provided regarding whether and to what extent formative research and testing were conducted—a limitation common to many other published mHealth-based behavioral change interventions to date. To advance the science in this area, recent recommendations and guidelines call for detailed reporting of the types of methods used in each phase of the iterative design process [18-20].

Human-centered design (HCD) is an approach that can be applied to guide researchers in the reporting of these iterative design processes [20,21]. In particular, HCD engages participants in defining their unmet needs and designing solutions to address them. Within the context of health care and biopharmaceutical industries, HCD uses a patient-centered approach that emphasizes the human perspective, in addition to including criteria such as technological feasibility and economic viability when designing an intervention solution [22].

The application of HCD in the field of health and disease symptom management has been growing in recent years [21]. To date, HCD has been used to design interventions addressing a range of conditions and issues, including chronic obstructive pulmonary disease, diabetes, caregiver stress, and posttraumatic stress disorder and for a range of users (eg, patients, HCPs, and caregivers) [21]. Interventions based on an HCD approach have demonstrated greater satisfaction, usability, and effectiveness than traditional ones [21]. A defining feature of HCD is contextual inquiry—a method in which users are observed and questioned in their own environments to obtain rich information about practices, the social, technical, and physical environments, and user tools. This method can be particularly useful for understanding daily patient experiences and leveraging those insights to inform the design of tools to support patients’ self-management.

### Objectives

This paper describes the application of an HCD approach to guide the development of a research protocol to inform the design of a digital intervention for RA symptom management in the context of a biotechnology company. Specifically, we aim to describe the steps in the conceptualization process, the purpose of each step, and the corresponding methods and data sources used. We seek to contribute to the body of knowledge regarding the methods for designing mHealth tools to support RA symptom management and medication adherence in the real world.

## Methods

We used an HCD approach to develop an intervention to assess RA-related symptoms and support treatment adherence. Signature features of HCD include the use of collaborative, multidisciplinary teams, an iterative design process involving rapid prototyping of solutions, and attention to the contexts in which the solution will be delivered [23]. The HCD approach is characterized by three main phases: (1) understanding, (2) ideation, and (3) implementation. Understanding involves exploring the dimensions, depth, and complexity of the opportunity or problem to be addressed. Ideation consists of generating, developing, and testing ideas or solutions for the identified problem. Finally, implementation involves rapid prototyping of ideas to produce solutions (eg, products and services), which are further refined via a series of subsequent iterations and feasibility assessments, including limited piloting or scaling-up efforts [23,24].

### Ethical Considerations

No ethics board review was sought because the institution sponsoring this research (Amgen, Inc.) classified this research as market research. Amgen did not require ethics committee approval for healthcare market research undertaken by professional market researchers on behalf of pharmaceutical or medical device companies where such research is conducted by professional market researchers in accordance with the legal and ethical guidelines such as those issued by the British Healthcare Business Intelligence Association (BHBIA) except where otherwise required by law. Consistent with BHBIA ethics guidelines, the authors acquired informed consent of study participants.

## Results

### Overview

We applied the HCD framework by mapping the key research objectives to each phase. In addition, we identified the need to add a postimplementation phase as well. The purpose of this postimplementation phase was to inform future improvements to the digital intervention post launch. A summary of the steps of the framework, the purpose of each step, and the methods and data sources used for each phase are presented in Multimedia Appendix 1 and Table 1. Throughout the course of the project, a multidisciplinary team was leveraged to conduct various analyses to support the HCD process. The team consisted of experts in qualitative methods, health services research, design research, and digital health technology. In addition, as part of the iterative HCD process, patients and providers were integrated into the co-design and concept pretest phases.

**Table 1:** Data sources used to guide development of rheumatoid arthritis (RA) symptom management and treatment adherence intervention conceptualization.

Source	Objective	Methodology
Health care claims administrative data	To describe the size and characteristics of entire RA biologics nonadherent population	Conduct secondary data analysis of longitudinal patient data on adherence and persistence
Disease registry or medical chart data	To understand the rationale for nonadherence	Analyze aggregate RA registry data from people with RA who had initiated biologic treatments
Electronic health record	To characterize the different subtypes of patients based on rationale for drop, switch, or holiday and response rate	Analyze patient-level electronic health records for people with RA taking biologics
Patient social listening	To understand the underlying drivers of adherence to biologic treatment based on analysis of content of patient conversations with other patients	Scan social media for Patients with RA’ conversations based on a list of keywords
HCP^a^-patient conversations and digital ethnography	To understand patient conversations with physicians and underlying drivers of adherence	Analysis of physician-patient with RA conversations (audio and transcripts) with redacted physician-client information
Call center	To gain insight into questions and concerns that patients have with treatment	Analysis of redacted Biologics: support call center conversations between nurses and patients
HCP ethnographic research	To gain insight into physician or office needs in helping to set RA treatment expectations and to support adherence	Conduct facility-based in-depth interviews with rheumatologists, including a simulated interaction with an actor-patient incorporating expectation-setting materials
Patient with RA ethnographic research	To gain insight into Patients with RA’ experiences with using biologics and needs regarding support for adherence	Conduct interviews with patients with RA on biologics treatment, including at-home exercises, quantitative surveys and follow-up telephone in-depth interviews

^a^HCP: health care professional.

### Phase I: Understanding

The understanding phase in the framework consists of a review and synthesis of a variety of different primary and secondary data sources to define the problem and to identify and frame the unmet needs to be addressed (Multimedia Appendix 1).

A range of primary and secondary data sources were identified and analyzed to characterize and explicate the rationale for medication nonadherence behaviors among patients diagnosed with moderate to severe RA who were being treated with a biologic product (Table 1).

We included four types of secondary data sources in the analysis: (1) administrative health care claims, (2) electronic health care records, (3) patient-level chart data, and (4) social media data. Primary data collection involved the use of ethnographic methods to obtain in-depth insights from both HCPs and people with RA.

Findings from this phase were instrumental in informing the problem definition for the intervention to address. Analysis and synthesis were conducted to identify distinct behavioral profiles related to adherence to RA biologic treatment, and criteria of actionability and measurability were used to select the profiles on which to intervene. Specifically, the profiles that were selected were ones in which there were defined behavioral objectives for both patients and providers, identifiable timing parameters for delivering an intervention, an understanding of the barriers to the desired behaviors (eg, *cloud of doubt* in patients’ minds regarding whether the treatment was working or was continuing to work), and a defined critical *turning point* after which treatment adherence would be likely to decline (ie, 3-month mark posttreatment initiation).

The findings from this study enabled the research team to formulate a working hypothesis to guide the next steps in formative development.

### Phase II: Ideation

The ideation phase consisted of three components: (1) a cocreation activity with patients and HCPs, (2) a patient journaling exercise, and (3) a literature review. The purpose of the cocreation activity was to inform the design team regarding patient and HCP needs and expectations during the patient-provider conversation to address barriers to medication adherence. Specific cocreation activities consisted of role plays with a small sample of rheumatologists, nurse practitioners, and patients in the clinic setting. In addition, patient journaling activities were used to gain a deeper understanding of patients’ expectations regarding their treatment, how they managed their weekly routine, and the key differentiating factors between adherent and nonadherent patients. In addition, a literature review was conducted to identify previous e-diary interventions in patients with RA and validated PROMs to determine how (visual analog scales) and when the PROMs should be sent.

This phase resulted in a set of recommendations for an initial concept design. This concept was refined and tested in the implementation phase to address questions related to the understandability of the content and the optimal delivery of the intervention (ie, timing, frequency, and cadence).

### Phase III: Implementation

The implementation phase consisted of (1) additional formative research to refine the intervention concept and (2) prototype-testing of the selected intervention to assess how to integrate it into patients’ lives and to assess whether the content was understandable and acceptable.

#### Formative Research

The formative proof-of-concept research was conducted on a sample of patients with RA on treatment. The primary goals of the formative study were (1) to understand patient reactions to receiving multiple daily SMS text messages to assess the state of their RA symptoms and (2) to determine whether patients comprehended the content of the SMS text messages and found them to be useful. In total, 10 patients participated in the formative study for up to 4 weeks. The secondary goal was to identify a data structure that would enable the comparison of longitudinal symptom data. Determining the optimal time of day (if any) to prompt for reports of pain, fatigue, and length of morning stiffness would inform design decisions regarding how to capture and visualize the data in the next iteration.

Methods included 12 daily PROM surveys conducted via SMS text messages and weekly 30-minute patient interviews conducted via telephone. Weekly interviews were conducted to elucidate patient comprehension of the data, interpretation and utility of changes in the reported data over time, perceptions of the relationship between changes in reported data and current pain, fatigue, and morning stiffness, and feedback regarding the receipt of messages based on data. In addition, analyses were conducted to identify patterns in patients’ responses to PRO text messages.

A protocol was developed to determine the optimal time for delivery of each PRO assessment [25]. The protocol probed for frequency of text messaging for pain assessment (randomly scheduled vs predetermined time points) and the type of pain scale to use (eg, 0-10 scale with 0=no fatigue to 10=totally exhausted). Similar questions were asked regarding the frequency and periodicity of the assessment and the preferred scale for measuring morning stiffness and fatigue.

The outcome of this phase included findings related to patients’ (1) perceptions of the meaningfulness and usefulness of the data; (2) preferences for the timing, frequency, and cadence of the messages; and (3) the need for support in interpreting and responding to the PROs (pain, fatigue, and morning stiffness). The results of the formative phase yielded information regarding both aspects of the intervention prototype design and intervention impact. Table 2 presents examples of the types of findings at the completion of this phase.

On the basis of the learnings, the intervention was revised to reduce the number and timing of PRO assessments, to include the provision of a biweekly symptom report that visualized PRO data over time, and to send motivational and feedback messages to promote sustained patient engagement. The revised intervention then underwent concept testing in a new sample of patients with RA.

**Table 2:** Types of findings from the formative study.

Domain and specific constructs		Example of types of findings
**Intervention delivery**		
	Patients’ preferences for the timing, frequency, and cadence of the messages	Ideal number of messages Preferences for a message schedule Preferred amount of time to respond to PROM^a^-related messages
	Type of support to interpret and respond to the PROMs	Understanding of different visual representations of their own data Key elements to include in the data visualization Comprehension and interpretation of how to respond to PROM messages (eg, whether it should be based on the last hour or the moment the message was received) Feedback on motivational messages
**Perceived impact**		
	Patient perceptions of the meaningfulness and usefulness of the data	Perceived potential impact of the intervention including Awareness of short- and long-term changes in symptom severity Usage of their data to have informed discussions with their rheumatologist regarding their symptoms Perceived usefulness to support medication adherence

^a^PROM: patient-reported outcome measure.

#### Prototype Testing

Following the formative phase, 2 working prototypes of the concept were pretested on patients with RA. The first prototype-testing was intended to capture patient feedback with regard to the modified intervention in a sample of biologics-naive patients with RA (prototype test 1). For example, patients provided feedback on the frequency of SMS text message requests to report symptoms and their willingness to engage in a 12-week program. The second prototype test sought to elicit feedback on the presentation of data in the symptoms journals when presented in different layouts in a subset of patients with RA who had completed the initial concept testing (prototype test 2).

Specific objectives of the prototype-testing 1 and 2 were to understand the use of a digital journal to help monitor or manage patients’ disease conditions; learn if participants were able to understand and interpret the data presented (ie, the data visualizations) in 2 different presentation layouts—the original graph views and metaphoric landscape views that reflected a patient’s data (eg, high levels of pain would create steep mountains vs low pain levels would create a green meadow); gauge participants’ opinions on the various elements in the 2 new presentations—data visualizations and the surrounding templates; and appraise the participants’ point of view on system components.

Feedback on the presentation of data in the journal was used to evaluate the best approach to visualize data in a graph format and to learn the merits of a graph versus a metaphoric view of data visualization.

Findings from this phase helped clarify the value proposition of the intervention for patients and the understandability and preferences for data visualizations. Specifically, findings were used to select the visual presentation of the data and guidance on data interpretation (eg, including question scaling) and to inform the final version of the intervention.

### Phase IV: Postimplementation Assessment

A postimplementation assessment was conducted to assess patients’ experiences using the intervention in a real-world setting. Specific objectives included understanding how the intervention was being used by both patients using a biologic product to treat RA symptoms and prescribers in clinical practice and how it could be changed to enhance its usefulness to patients. To address these objectives, an SMS text messaging–based survey was delivered to past intervention participants. In addition, interviews were conducted with 20 patients with RA and 10 rheumatologists who participated in the intervention. These surveys included conceptual stimuli to assist participants in *thinking aloud* and verbalizing their thoughts.

Results from a thematic analysis of the results from this phase of the research were intended to yield information regarding patient use patterns and descriptions of their experiences interacting with both the digital journaling tool and the weekly graphical output. Information was also obtained regarding both HCP and patient perceptions of the benefits of the intervention and their recommendations for its enhancement. Patients were asked to describe how the intervention affected their self-management of symptoms during the periods between HCP visits and how the intervention influenced their communication with their HCPs. These descriptions included the *emotional dimension* of their care experience (ie, their perceptions of their care and the feelings that the care experience evoked in them) while participating in the intervention.

## Discussion

### Principal Findings

The field of mHealth intervention research is growing rapidly. Mobile phones are both easily accessible and widely used, and they possess an ever-expanding array of features and technical capabilities [26,27]. Therefore, their use as a platform for health interventions can only be expected to increase in the coming decades. Currently, research guidelines are calling for a systematic approach to documenting the iterative formative design processes to contribute to the evidence base for effective patient-centered mHealth interventions and to support the effective application of such interventions in real-world settings [18,19].

HCD offers a well-tested approach for addressing this gap and for helping develop an applied framework for use in the biopharmaceutical industry context. The adapted framework emerged from an inductive process derived from the development and pilot testing of a digital-based intervention to support symptom management among patients living with RA. The framework featured four separate phases: (1) understanding the dimensions and complexities of symptom management and treatment nonadherence among patients with RA, (2) intervention concept ideation, (3) iterative prototype development of the intervention and pretesting via piloting, and (4) postimplementation assessment.

Applying an HCD-based approach demands commitment to conducting in-depth formative research and an iterative approach to developing interventions. This is because an HCD approach emphasizes understanding of (1) the *context* of chronic disease management, which is a critical consideration for developing effective intervention strategies; (2) the *acceptability* of the proposed intervention to the intended recipients and those involved in its implementation; (3) *the demand* for and value of the intervention, as determined by piloting the use of selected intervention activities; and (4) the *implementation requirements*. In addition, using iterative design cycles, HCD guides ongoing intervention design in response to the circumstances and constraints encountered in real-world application [28].

Our goal in sharing this protocol is to increase the transparency of mHealth design efforts, thus aligning with recent legislative imperatives such as the 21st Century Cures Act [29]. A range of different frameworks are available to guide such efforts, including those that combine both human-centered and sociotechnical design considerations [30].

The importance of leveraging frameworks that consider the complexities of chronic disease management, the variety of stakeholders involved, and practical guidance for developing effective digital health solutions has been acknowledged [30]. Similar to our findings, van Gemert-Pijnen et al [30] emphasize the importance of systematic evaluation incorporating multiple stakeholders to ensure that solutions are user-informed, are fit for context, and add value.

Although such a comprehensive, holistic approach is recommended for use in future real-world applications of this type, the exact framework used is less important than the fact that a framework itself was applied to guide the development process. The application of a framework is critical for enabling a systematic approach to industry contributions to building, testing, and disseminating digital health interventions that help generate evidence regarding effective approaches in real-world settings.

### Limitations

Arguably, a limitation of the HCD approach is that it excludes the postimplementation experience. To address this, we added a postimplementation assessment phase to our framework. However, our assessment was limited in scope, both in terms of outcomes evaluated and the duration of follow-up. Further work is needed to strengthen the postimplementation assessment phase so that the degree to which the intervention was adopted and sustained over time in the real-world context can be monitored and evaluated.

Another limitation concerns the use of real-world evidence and data analytics; there is a need for further guidance on a systematic approach to identify and evaluate the range of real-world data sources that might be appropriate for use.

### Conclusions

The application of an HCD approach in a biotechnology industry setting helped inform the development of a research protocol for designing a digital health intervention for patients with moderate to severe RA. The application of this framework provided a structured road map for obtaining comprehensive, actionable insights regarding patients’ daily experiences living with RA, the context of and barriers to symptom management, and treatment adherence from the perspectives of both the patient and HCPs. Collectively, such information helped directly inform the design of the intervention and increased the likelihood that it would prove acceptable, feasible, engaging, and impactful when implemented under real-world circumstances.

## Appendix

Multimedia Appendix 1 Framework overview of the development of a rheumatoid arthritis symptom management and treatment adherence digital intervention.

## Abbreviations

HCD: human-centered design
HCP: health care professional
mHealth: mobile health
PRO: patient-reported outcome
PROM: patient-reported outcome measure
RA: rheumatoid arthritis
