# Supplementary material for: Development of a Digital Health Intervention for Rheumatoid Arthritis Symptom Management in a Biotechnology Industry Context: Protocol for the Application of a Human-Centered Design Framework
Source: JMIR Res Protoc. 2022 Mar 22;11(3):e16430. doi: 10.2196/16430 (PMC8984827; doi:10.2196/16430)
Supplement: Multimedia Appendix 1 [file resprot_v11i3e16430_app1.docx]

**Multimedia Appendix 1.** Framework overview of the development of a rheumatoid arthritis symptom management and treatment adherence digital intervention.

| Summary | Framework phase and steps | | | | |
| --- | --- | --- | --- | --- | --- |
|  | Understanding | Ideation | Implementation | | Postimplementation assessment |
|  | Problem identification | Concept design | Formative research | Prototype-testing | Evaluation of the concept post-pilot |

|  |
| --- |

| Description and purpose | - Define problems and opportunities that interventions should address | - Describe patient and HCP^a^ needs for RA^b^ management - Identify intervention concepts | - Assess patient reactions to the concept developed and understandability of its content | - Pilot-test the prototype concept to assess patient perception of intervention delivery and gain feedback on printed journal data visualization | - To determine patient and HCP experiences using the prototype intervention |
| --- | --- | --- | --- | --- | --- |
| Phase duration | - Approximately 12 weeks | - Approximately 4 weeks | - Approximately 4 weeks | - Approximately 20-24 weeks | - Approximately 3 weeks |
| Specific objectives | - Better understand drivers of nonadherence for RA biologic - Develop profiles of patients who are not adherent because of perceptions of efficacy and/or the difficult routine - Define the unmet need to be addressed from a patient’s perspective | - Develop concepts to facilitate setting treatment efficacy expectations between HCPs and patient by building testing stimuli and cocreating in a roleplay with patients and HCPs in the doctor’s office - Identify potential patient-reported outcomes to be included | - To improve and refine intervention from feedback - Assess patient reactions to texts for RA management - Understand patient reactions to receiving texts to assess the state of their RA symptoms through multiple daily self-reported measure assessments (pain, fatigue, and morning stiffness) - Ensure that patient understanding of the texts received is clear and inoffensive | - Capture patient feedback about the presentation of data in the paper journals on both graph and landscape views - Understand behavioral triggers to appropriately encourage patient journaling over time | - Understand potential improvements of intervention - Understand HCP needs for intervention, as it was designed as a patient program - Understand how the program could be expanded to meet the needs of other therapeutic areas such as oncology |
| Research questions addressed | - What types of behaviors lead to nonadherence? - When do nonadherent behaviors take place? - Why do patients behave this way? - What is the unmet need to be addressed and how is it expressed in *human terms*? | - Does digital intervention address patients’ concerns regarding whether the treatment is working or still working (ie, *cloud of doubt*)? | - How well do patients comprehend the data? - Can patients accurately interpret and use changes in the reported data over time? - What are the patient perceptions of the relationship between changes in reported data and current pain, fatigue, and morning stiffness? | - How do patients use a journal to help manage their Illness (RA)? - Are participants able to understand and interpret the data presented? - What are participants’ opinions on the various elements in the 2 new presentations? - Do participants understand the meaning of the landscape metaphor/visualization? - What are patients’ points of view on the system components? | - Patients:   - How are patients using the intervention in the field?   - How do patients benefit by using the intervention?   - How can the intervention be changed to enhance its usefulness for patients?   - What did patients learn about managing RA or symptoms that they may not have already known before using the program? - Rheumatologists:   - How did rheumatologists use the intervention in the field?   - How did rheumatologists benefit when patients used the intervention?   - How can the intervention be changed to enhance its usefulness for rheumatologists?   - What were some initial expectations for using the intervention with patients? |
| Sample size | - N/A^c^ | - n=4 rheumatologists, n=3 nurses, and n=7 patients | - n=10 patients | - n=13 Patients with RA | - n=20 intervention experienced patients and n=10 intervention experienced rheumatologists |
| Methods | - Data analytics - Secondary data analysis - Synthesis of research | - Patient prework: journey map - In-depth interviews: HCPs and patients; cocreation activity/participatory design to develop stimuli concepts around the problem of *setting expectations* - Role play *setting expectations:* conversation with 3 HCPs and 7 patients using drafted stimuli - Follow-up in-depth interviews: HCP-patient dyad, HCPs, and patients individually | - In-depth interviews: biologics-naive Patients with RA who used the intervention for 4 weeks | - Patients with RA pilot-tested the working prototype from 3 weeks to 11 weeks - Web-based surveys - Individual semi-structured qualitative interviews | - An SMS-based survey was delivered to past intervention users - In-depth interviews: individual semi-structured qualitative interviews; over the phone and lasted between 60 and 90 min; using conceptual stimuli to assist participants to *think aloud* and verbalize their thoughts - Patient ratings of the digital intervention using a 5-point Likert scale |
| Data sources | - EMR data - Patient-level data - Social media platforms - Digital ethnography | - Co-creation activity output: journey map and refined stimuli - In-depth interview audio/transcripts | - In-depth interviews audio/transcripts | - Prototype test 1: web-based surveys with 27 bio naive Patients with RA who used the intervention for up to 12 weeks - Prototype test 2: interviews with 13 patients who completed test 1 | - In-depth interviews - Surveys |

^a^HCP: health care professional.

^b^RA: rheumatoid arthritis.

^c^N/A: not applicable.
